# Impact of *cis*-acting elements’ frequency in transcription activity in dicot and monocot plants

**DOI:** 10.1007/s13205-015-0305-6

**Published:** 2015-05-13

**Authors:** Ghada A. Abu El-Heba, Gihan M. Hussein, Inas F. Fahmy, Sara M. Abdou, Asmaa Faisal, Omnia Taha, Naglaa A. Abdallah

**Affiliations:** Department of Nucleic Acid and Protein Structure, Agricultural Genetic Engineering Research Institute (AGERI), ARC, Giza, Egypt; Department of Gene Transfer, Agricultural Genetic Engineering Research Institute (AGERI), ARC, Giza, Egypt; Department of Microbial Molecular Biology, Agricultural Genetic Engineering Research Institute (AGERI), ARC, Giza, Egypt; Department of Genetics, Faculty of Agriculture, Cairo University, Giza, Egypt

**Keywords:** *Agrobacterium* infiltration, GUS fluorometric assay, Putative promoters, Recombinant DNA technology, TYLCV, WmCSV

## Abstract

The production of new cultivars via recombinant DNA technology is important in applied agriculture. Promoters play fundamental roles in successful transformation and gene expression. Fragments of the upstream regulatory region of the movement protein gene of the *Tomato yellow leaf curl virus* (TYLCV; two fragments) and *Watermelon chlorotic stunt virus* (WmCSV, two fragments) and one fragment of the coat protein putative promoter of TYLCV (CPTY-pro) were isolated to assess their abilities to drive expression in monocot and dicot plants. We used bioinformatic analyses to identify tentative motifs in the fragments. The five promoter fragments were isolated, fused with the *GUS* reporter gene, and transformed into tomato, watermelon, and rice plantlets via *Agrobacterium* infiltration. *GUS* expression driven by each putative promoter was analysed using histochemical and fluorometric analyses. In both dicots and the monocots, the highest level of GUS expression was obtained using a truncated regulatory region from TYLCV (MMPTY-pro) followed by a truncated regulatory region from WmCSV (MMPWm-pro). However, the corresponding full-length fragments from TYLCV and WmCSV showed essentially equivalent expression levels in the fluorometric GUS assay compared with the enhanced *Cauliflower mosaic virus* e35S-pro. In addition, CPTY-pro showed no expression in either the dicots or the monocot. This study demonstrated that MMPTY-pro and MMPWm-pro may be useful as plant promoters.

## Introduction

In the last decades agriculture was considered to be biological machinery. With accumulative information of genetics, plant breeders have enhanced crop quality and yield. In this century recombinant DNA technology facilitated the characterization and isolation of valuable genes that can be introduced into living organisms to obtain new traits with improved qualities.

Gene expression levels and patterns depend on the presence or absence of *cis*-regulatory elements in their promoter regions. Expression can be monitored experimentally using a reporter gene under control of the putative promoter. Promoters can be constitutive, inducible, or tissue-specific. Some geminivirus promoters are able to drive constitutive gene expression in transgenic plants, while others are subject to regulation. For example, the CP promoter from *Tomato golden mosaic virus* (TGMV) is active in both phloem and mesophyll cells in the presence of a transcriptional activator protein (TrAP).

*Tomato yellow leaf curl virus* (TYLCV) is a member of the genus *Begomovirus* in the family *Geminiviridae* (Briddon et al. 1996). Its monopartite single-stranded genome encodes five proteins from both the virion (V) and complementary (C) strands: C1, C2, C3, V1, and V2. V1 and V2 are late-expression genes that code for the viral pre-coat and coat (CP) proteins, respectively; V2 expression is transactivated by the C2 protein. C1, or REP, is a replication protein that recognizes the origin sequence (ori) in the viral genome (Laufs et al. 1995). C1 can positively regulate expression by binding to the viral replication enhancer protein, C3 (Settlage et al. 1996; Castillo et al. 2003). C2 is involved in CP expression regulation (Dry et al. 2000) and plays a role in viral systemic spread in plants.

The last open reading frame in the group of early-expression genes is c4-ORF, which encodes a movement protein (MP) that is responsible for viral movement from cell to cell through plasmodesmata. We previously reported that gene was highly expressed (Abu El-Heba et al. 1999), so we decided to isolate and evaluate the expression efficiency of its promoter and compare it with another geminivirus MP promoter and the widely used *Cauliflower mosaic virus* e35S promoter as a control.

We chose to isolate the MP promoter from the *Watermelon chlorotic stunt virus* (WmCSV), a bipartite geminivirus (Lecoq et al. 1994; Dafalla et al. 1998; Kheyr-Pour et al. 2000). Segment A of its genome contains the same genes as the TYLCV genome, except the MP BC1, which is incorporated within intergenic region of segment B (Orozco and Hanley-Bowdoin 1996).

In this study, the putative MP promoters of TYLCV (MP-TYLCV) and WmCSV (MP-WmCSV) were tested for their effects on the expression of the β-glucuronidase (*GUS*) reporter gene. *Agrobacterium* infiltration was used to transform dicot and monocot plants with a promoter–*GUS* construct to monitor promoter activity. Both promoters showed great promise for recombinant protein production.

## Materials and methods

### Plant material

To study the isolated promoter fragments, watermelon (*Citrulluslanatus*; Cucurbitaceae) cv. Giza1 and tomato (*Lycopersicon esculentum*; Solanaceae) cv. CastleRock were used as model dicots, while rice (*Oryza sativa*; Poaceae) cv. Swat II was used as a monocot model. 5-day-old hypocotyledonous discs of watermelon and tomato were cultured as explants on MS medium (Murashige and Skoog 1962) supplemented with 1 mg/L 6-benzylaminopurine (BAP), 0.25 abscisic acid and 5 mg/L AgNO_3_. Rice callus was initiated from seeds on MS medium containing 2 mg/L of 2,4-dichlorophenoxyacetic acid for 1 month.

### Culture incubation conditions

All regeneration experiments were conducted in MS medium containing 30 g/L sucrose and 8.0 g/L agar, with a pH of 5.8 before autoclaving. All plant cultures were maintained in a growth chamber at 25 °C ± 2 under fluorescent lights (8/16 h dark/light).

### Cloning the putative TYLCV and WmCSV promoters

**Fig. 1:**
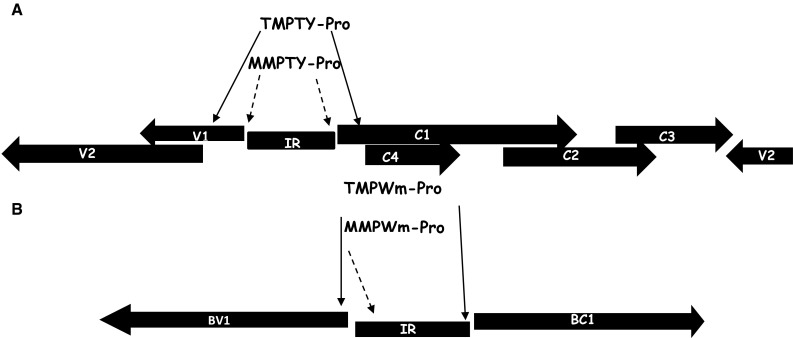
Schematic drawing of viral genomes. **a** *Tomato yellow leaf curl virus* genome organization. *V1* and *V2* are on the virion strand while *C1*, *C2*, *C3*, and *C4* (MP promoter) are on the complementary strand. **b** *Watermelon chlorotic stunt virus* DNA-B genome. *BV* the nuclear shuttle protein (NSP) is in the virion sense orientation, while BC1 (MP) is in the complementary sense. The *solid arrow* indicates the complete MP and CP promoter fragments (MPTY-pro and CPTY-pro) of TYLCV and the complete BC1 promoter regions of the WmCSV (MPWm-pro), while the *dashed arrows* indicate the shorter promoter fragments

Figure 1 depicts the locations and the directions of the promoter fragments in the TYLCV (3 fragments) and WmCSV (2 fragments) genomes. These fragments represent the MP gene promoters in both viruses as well as the CP promoter gene of TYLCV (Table 1). To facilitate cloning of the fragments, the restriction sites for *Hin*dIII and *Bgl*II were added to the 5′ and 3′ ends, respectively, of all MP promoters, and to the 3′ and 5′ ends, respectively, of CP promoters fragments.

**Table 1:** Constructs description for the five putative promoter fragments

Source	Promoter	Size (bp)	Construct name	Orientation
TYLCV	CPTY (full length of CP promoter)	570	pCPTY	+Strand
TYLCV	TMPTY (full length of MP promoter)	570	pTMPTY	−Strand
TYLCV	MMPTY (minor length of MP promoter)	300	pMMPTY	−Strand
WmCSV	TMPWm (full length of MP promoter)	750	pTMPWm	−Strand
WmCSV	MMPWm (minor length of MP promoter)	550	pMMPWm	−Strand

For the putative MP promoters of both TYLCV and WmCSV, we designed specific primers to amplify both the entire putative promoter, respectively, called TMPTY-pro (~570 bp in size, specific primers PGLH and PGLB) (accession no. KP419702) and TMPWm-pro (~750 bp, primers PEH1 and PEB1) (accession no. KP657700), and a smaller “minimal” fragment, respectively, called MMPTY-pro (~300 bp, PGSH and PGSB) and MMPWm-pro (~550 bp, PEH2 and PEB2). One set of specific primers, CPB and CPH, was used to amplify the CP promoter (CPTY-pro; ~570 bp in size). Table 2 showed the oligonucleotide sequences of all primers sets used to amplify the promoter fragments.

**Table 2:** Oligonucleotide primers used to amplify different sized promoter fragments from geminiviruses

Oligonucleotides primers	Sequence	Restriction site added
PGLH	5′-CCC*AAGCTT*AGTCACGGGCCCTTACAAC-3′	*Hin*dIII
PGLB	5′GA*AGATCT*GGAGATGTGGTTCCCCATTC-3′	*Bgl*II
PGSH	5′-CCC*AAGCTT*ATTGCAAGACAAAATACTT-3′	*Hin*dIII
PGSB	5′- GA*AGATCT*ATTTTAAATAAACGAGGCAT-3′	*Bgl*II
CPB	5′-GA*AGATCT*AGTCACGGGCCCTTACAAC-3′	*Bgl*II
CPH	5′-CCC*AAGCTT*GGAGATGTGGTTCCCCATTC-3′	*Hin*dIII
PEH1	5′-CCC*AAGCTT*GGGACGTACGTCCCGTCACA-3′	*Hin*dIII
PEB1	5′-GA*AGATCT*TCTCCGTTCTTCCACAGGACC-3′	*Bgl*II
PEH2	5′-CCC*AAGCTT*AATATTATAGGATGGCC-3′	*Hin*dIII
PEB2	5′-GA*AGATCT*TCTCCGTTCTTCCACAGGACC-3′	*Bgl*II

Plasmid pTYNA101 (Abdallah et al. 1993) containing the full genome of TYLCV-Egyptian strain (Eg) was the template for amplifying the different sized promoter fragments of TYLCV. Restriction-digested **(***Hin*dIII–*Bgl*II) fragments were individually cloned into the plasmid pMONRTG (Liu 2003) from which the 35S promoter had been excised, creating a *GUS* reporter gene under the control of the promoter fragment, i.e. two sizes of the MP promoter and one size of the CP promoter. The three pro::*GUS* constructs were excised using *Hin*dIII and *Eco*RI and ligated into a modified pCAMBIA1390 binary vector after excision of its 35S promoter.

Purified WmCSV preparations of the Sudan isolate (Kheyr-Pour et al. 2000) were used as a template for amplifying the different sized promoter fragments of WmCSV. Each restriction-digested (*Hin*dIII–*Bgl*II) promoter fragment was cloned into pMONRTG after the excision of its 35S promoter to yield a *GUS* reporter gene under the promoter’s control. The two constructs, TMPWm-pro::*GUS* and MMPWm-pro::*GUS*, were released from the vector using *Hin*dIII and *Bam*HI. These fragments were then ligated into a modified pCAMBIA1390 binary vector after excising its e35S promoter.

### Bioinformatics analyses

Subsequently, the five promoter fragments were sequenced using an ABI 3730xl sequencer (Applied Biosystems, Foster City, CA, USA), after cloning in pGEM-T Easy (Promega, Fitchburg, WI, USA). These sequences and that of the e35S promoter were analysed using Web Signal Scan (http://www.dna.affrc.go.jp/PLACE/signalscan.html) (Prestridge 1991).

### *Agrobacterium* strains and constructs

The five promoter constructs and pCAMBIA3301 were transformed into *Agrobacterium* strain GV3101 (Koncz and Schell 1986) by electroporation (Sukharev et al. 1992) for explant agroinfiltration. A construct with *GUS* under the control of the e35S promoter (GV3101::pCAMBIA3301) was used as a positive control for the *GUS* reporter gene. The five constructs each harboured the *GUS* gene in pCAMBIA1390 under control of a test promoter fragment: the large (GV3101::TMPTY-pro) or small (GV3101::MMPTY-pro) fragment of the TYLCV MP promoter; or the large (GV3101::TMPWm-pro) or small (GV3101::MMPWm-pro) fragment of the WmCSV-MP promoter. GV3101 was used a negative control.

### *Agrobacterium* infiltration

Agroinfiltration was carried out on watermelon, tomato, and rice explants. *Agrobacterium* cultures were grown overnight at 28 °C in liquid LB medium supplemented with 50 mg/L kanamycin and 10 mg/L rifampicin. When cultures reached about 0.8 at OD_600_, cells were harvested and diluted by re-suspending and incubating in MES buffer (10 mM MES/KOH, pH 5.6 and 10 mM MgCl_2_) for 2 h at room temperature to a final concentration of 1.0 at OD_600_. Tomato, watermelon, and rice explants were submerged separately in one of the seven *Agrobacterium*-suspension cultures and placed inside desiccators for infiltration at 200 mbar for 5 min. The infiltrated explants were incubated in the dark and assayed for *GUS* activity after 3 days.

### Detection of GUS expression

GUS expression was visually evaluated using the histochemical assay of Jefferson et al. (1987). The GUS reaction was performed 3 days post-agroinfiltration by incubating the samples with GUS buffer solution containing 1 mM 5-bromo-4-choloro-3-indolyl-β-glucuronic acid overnight at 37 °C. The blue colour was detected visually by the naked eye and using a light microscope (LEICA Icc 50 HD-DM750, Wetzlar, Germany) after bleaching the chlorophyll with 70 % ethanol.

### Quantitative GUS assay

A quantitative fluorescent assay was performed to evaluate *GUS* expression using the substrate 4-methylumbelliferylgalactopyranoside (MUG) and the FluorAce™ β-glucuronidase Reporter Assay Kit (Bio-Rad, Hercules, CA, USA). Protein was extracted from each sample, with five replicates for each putative promoter in each type of explant (tomato, watermelon and rice). Each sample (5 µg) was incubated with 1 mM MUG buffer at 37 °C for 15 min. Stop buffer (1×) was used to terminate the reaction. The fluorescent molecule 4-methylumbelliferone (4MU) was released by hydrolysis of the fluorogenic substrate by GUS. Fluorescence of 4MU was measured with a Versafluor fluorometer (Bio-Rad) using an emission wavelength of 460 nm and an excitation wavelength of 360 nm. A fresh preparation of 100 nM 4MU was used as a calibration standard. GUS activity was then calculated as the production of 4MU from MUG in pmol/min/µg of protein. The mean GUS activity from the e35S promoter of pCAMBIA3301 was considered to be 100 % and used to standardize the activities from promoters in other constructs.

## Results

### Isolation, sequencing, and analysis of promoter fragments

Dissection analysis of the five promoter regions isolated from TYLCV and WmCSV can assist in identifying *cis*-acting signals and their interactions with *trans*-acting factors that contribute to eukaryotic gene expression. The isolated fragments were amplified using specific primers, cloned, and sequenced. The sequences were analysed for the presence of *cis*-acting elements that could affect transcription efficiency. The resulting motifs were counted and their loci recorded.

**Fig. 2:**
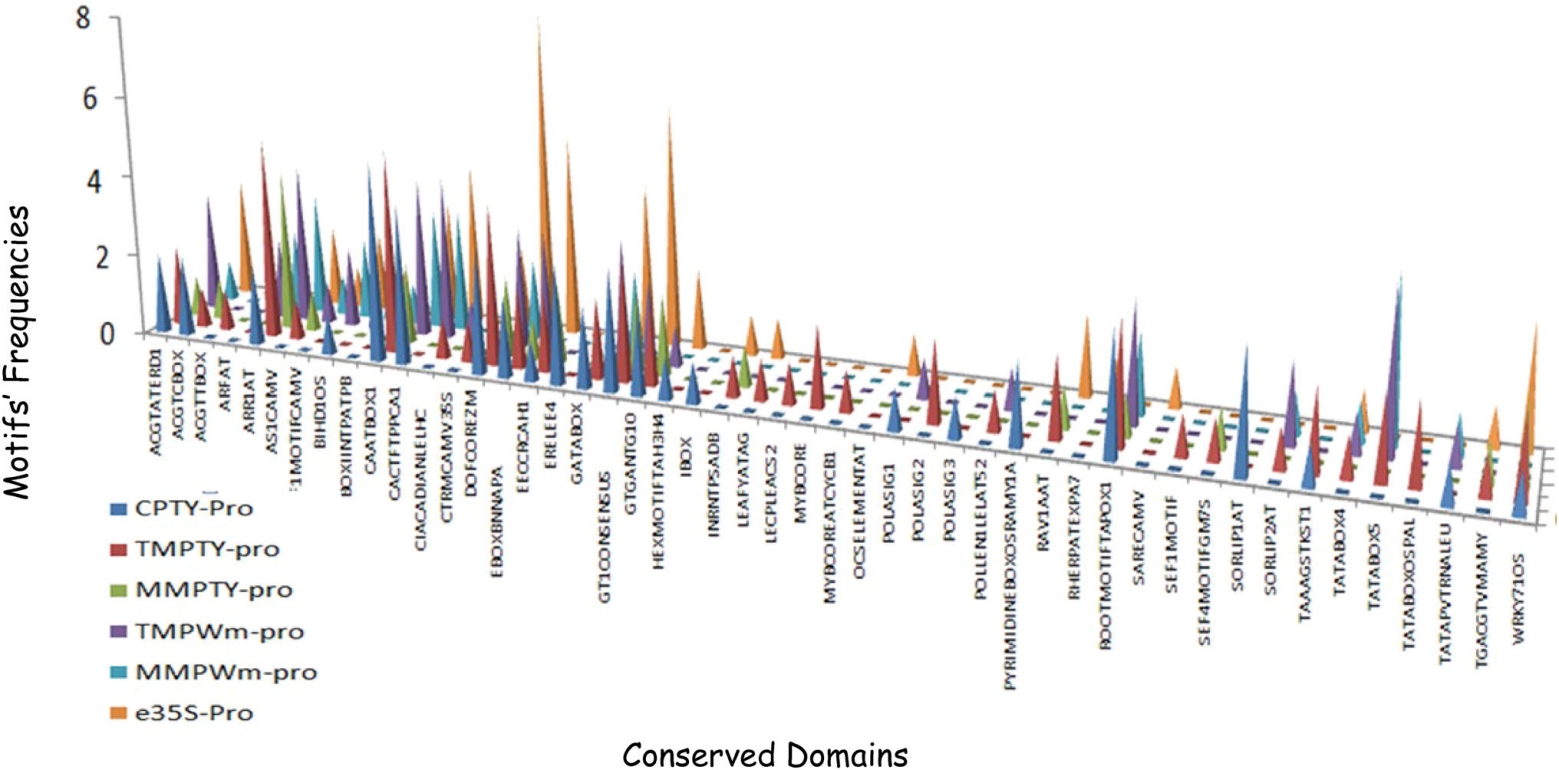
Conserved motif frequencies in putative promoters CPTY-pro, TMPTY-pro, MMPTY-pro, TMPWm-pro, MMPWm-pro and e35S-pro

**Fig. 3:**
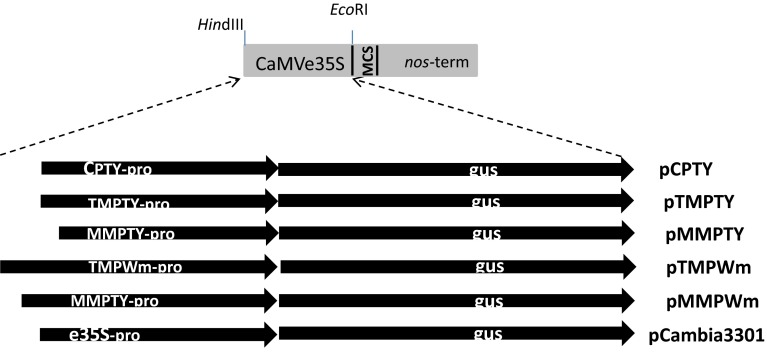
Cloning of the putative promoter fragments into modified pCambia 1390 binary vector by replacing the e35S promoter to drive *GUS* expression

The motifs in the five promoter regions and their frequencies were compared with those of e35S-pro (Fig. 2). To investigate the efficiencies of the regulatory regions, the fragments were cloned into a modified pCAMBIA1390 binary vector to replace the e35S promoter driving *GUS* expression (Fig. 3). Agroinfiltration was used to introduce the constructs into tomato, watermelon plantlets and rice callus to test the promoter efficiencies in dicots and a monocot.

### In silico identification of regulatory motifs and data analysis

In silico comparisons of the orthologous promoter fragments were performed using Web Signal Scan. Five conserved motifs were detected in all five putative promoters as well as the e35S promoter: ACGTATERD1 (5′-ACGT-3′), ARR1AT (5′-NGATT-3′) (Sakai et al. 2000), CAATBOX1 (5′-CAAT-3′), DOFCOREZM (5′-AAAG-3′) (Yanagisawa and Schmidt 1999), and GATABOX (5′-GATA-3′). Their positions and frequencies in our putative promoter fragments are shown in Table 3.

**Table 3:** Positions and the frequencies of the five conserved motifs present in CPTY-pro, TMPTY-pro, MMPTY-pro, TMPWm-pro, MMPWm-pro and e35S-pro

Domain	Consensus	CPTY-pro	TMPTY-pro	MMPTY-pro	TMPWm-pro	MMPWm-pro	e35S-pro
ACGTATERD1	ACGT	2 sites	2 sites	1 site	3 sites	1 site	3 sites
Position in URR		376, 410	376, 410	242	490, 726, 730	490	396, 407, 447
Percentage		4	4	6	6	3	5
ARR1AT	NGATT	2 sites	5 sites	4 sites	4 sites	3 sites	2 sites
Position in URR		587, 513	164, 288, 293, 391, 502	30, 154, 159, 257	111, 225, 306, 658	111, 225, 306	224, 424
Percentage		4	8	15	8	10	4
CAATBOX1	CAAT	5 sites	5 sites	2 sites	3 sites	2 sites	3 sites
Position in URR		99, 265, 390, 467, 572	67, 101, 261, 397, 578	127, 263	231, 434, 692,	231, 434	85, 180, 464
Percentage		9	8	7	8	10	5
DOFCOREZM	AAAG	3 sites	4 sites	2 sites	3 sites	2 sites	8 sites
Position in URR		73, 178, 300	85, 338, 344, 433	204, 210	21, 387, 598	21, 387	10, 46, 152, 174, 198, 271, 308, 313, 353, 389
Percentage		6	6	7	6	6	14
GATABOX	GATA	2 sites	2 sites	1 site	3 sites	2 sites	4 sites
Position in URR		106, 416	200, 607	66	257, 425, 642	257, 425	155, 262, 305, 434
Percentage		4	3	4	6	6	7

Bioinformatics analysis also revealed unique *cis*-elements in some fragments: CPTY-pro contained ERELEE4 (5′-AWTTCAAA-3′) at positions 266, 273, and 280 URR; HEXMOTIFTAH3H4 (5′-ACGTCA-3′) at position 376 URR; PYRIMIDINEBOXOSRAMY1A (5′-CCTTTT-3′) at positions 337 and 343 URR; and SORLIP1AT (5′-GCCAC-2′) at positions 583, 581, and 619 URR. Both TMPTY-pro and MMPTY-pro contained RAV1AAT (5′-CACCA-3′) at positions 154 and 611 URR and 20 URR, respectively; ARFAT (5′-TGTCTC-3′) at positions 405 and 445 URR. TMPWm-pro and MMPWm-pro both contained BOXIINTPATPB (5′-ATGAGAA-3′) at position 422 URR.

**Fig. 4:**
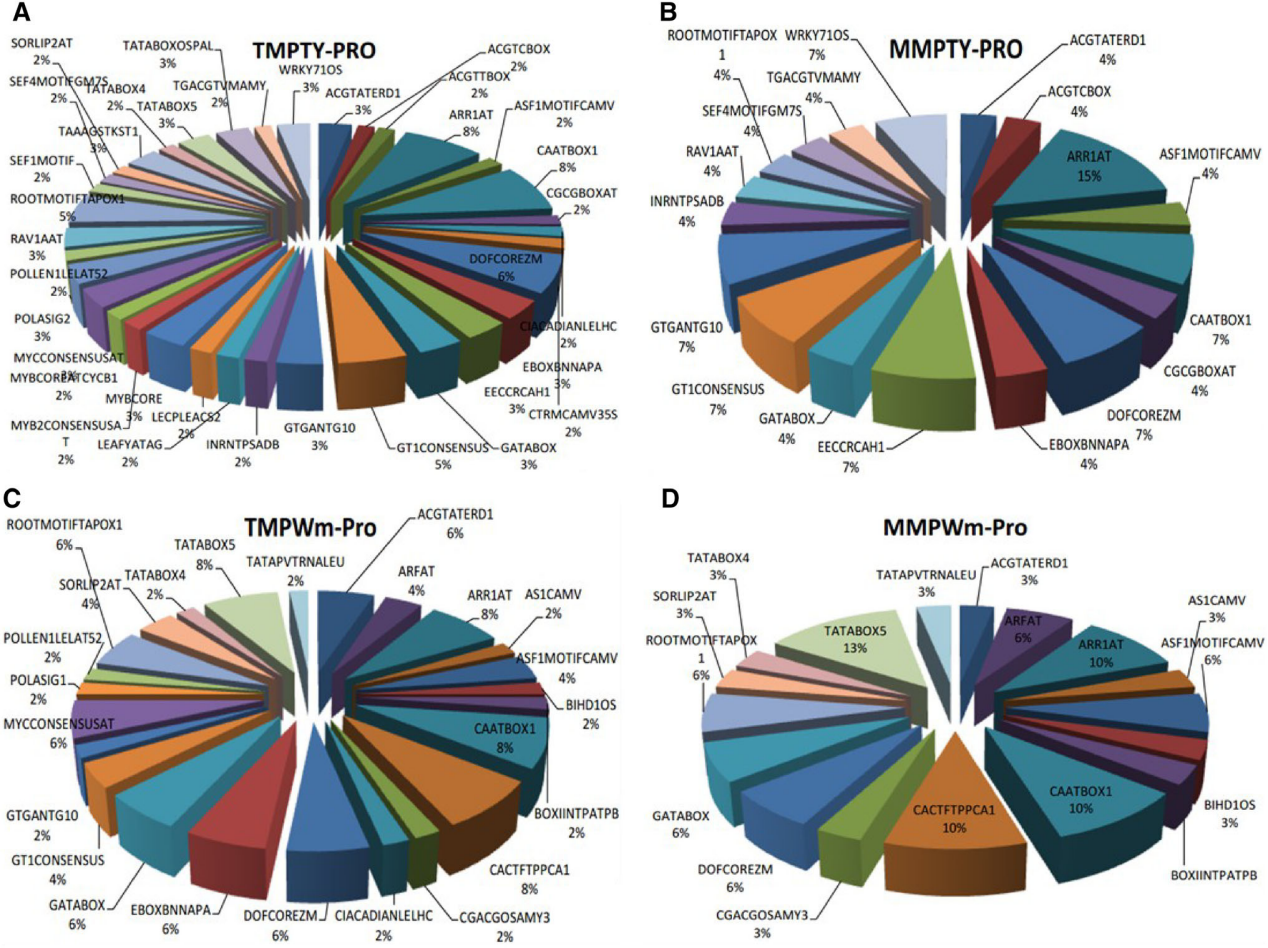
Composition of the promoters. **a** TMPTY-pro contained 59 conserved boxes; CAATBOX1 had the highest frequency (8 %). **b** MMPTY-pro comprised 26 conserved boxes, and ARR1AT was the most frequent (15 %). **c** TMPWm-pro had 48 conserved, with the most frequent being CAATBOX1 (8 %). **d** MMPWm-pro included 31 conserved boxes; TATABOX5 had the highest frequency (13 %)

In TMPTY-pro and MMPTY-pro (Fig. 4a, b), the ARR1AT represented 8 and 15 % of the promoter fragment lengths, respectively. The motifs CAATBOX1, DOFCOREZM, EECCRCAH1 (5′-GANTTNC-3′), GT1CONSENSUS (5′-GRWAAW-3′), GTGANTG10 (5′-GTGA-3′), WRKY71OS (5′-TGAC-3′) each represented 7 % of MMPTY-pro and 8, 6, 3, 5, and 3 %, respectively, of TMPTY-pro. Each of the other motifs comprised 4 % of MMPTY-pro and 2–5 % in TMPTY-pro. In TMPWm-pro and MMPWm-pro (Fig. 4c, d), the TATABOX5 comprised 13 % of MMPWm-pro and 8 % of TMPWm-pro. The motifs CAATBOX1, ARR1AT, and CACTFTPPCA1 (5′-YACT-3′) each made up 10 % of MMPWm-pro and 8 % of TMPWm-pro. Other motifs differed only slightly.

TATABOX4, TATABOX5, and TATABOXOSPAL were detected in TMPTY-pro, while they were completely absent in MMPTY-pro. TATABOX4 and TATABOX5 were recorded in TMPWm-pro and MMPWm-pro. Table 4 shows the distribution of TATA boxes within the five promoter regions and e35S-pro. Remarkably, CPTY-pro harboured the highest number of the *cis*-acting elements within its DNA sequence among the promoter regions (data are not shown).

**Table 4:** TATA box distributions and frequencies within CPTY-pro, TMPTY-pro, MMPTY-pro, TMPWm-pro, MMPWm-pro and the e35S promoter

Domain	Consensus	CPTY-pro	TMPTY-pro	MMPTY-pro	TMPWm-pro	MMPWm-pro	e35S-pro
TATABOX4	TATATAA	–	1 site	–	1 site	1 site	1 site
Position in URR		–	435	–	117	117	496
Percentage		0	2	0	2	3	2
TATABOX5	TTATTT	–	2 sites	–	4 sites	4 sites	–
Position in URR		–	117, 140	–	124, 169, 329, 368	124, 169, 329, 368	–
Percentage		0	3	0	8	13	0
TATABOXOSPAL	TATTTAA	–	2 sites	–	–	–	–
Position in URR		–	138, 535	–	–	–	–
Percentage		0	3	0	0	0	0

### Histochemical GUS assays

**Fig. 5:**
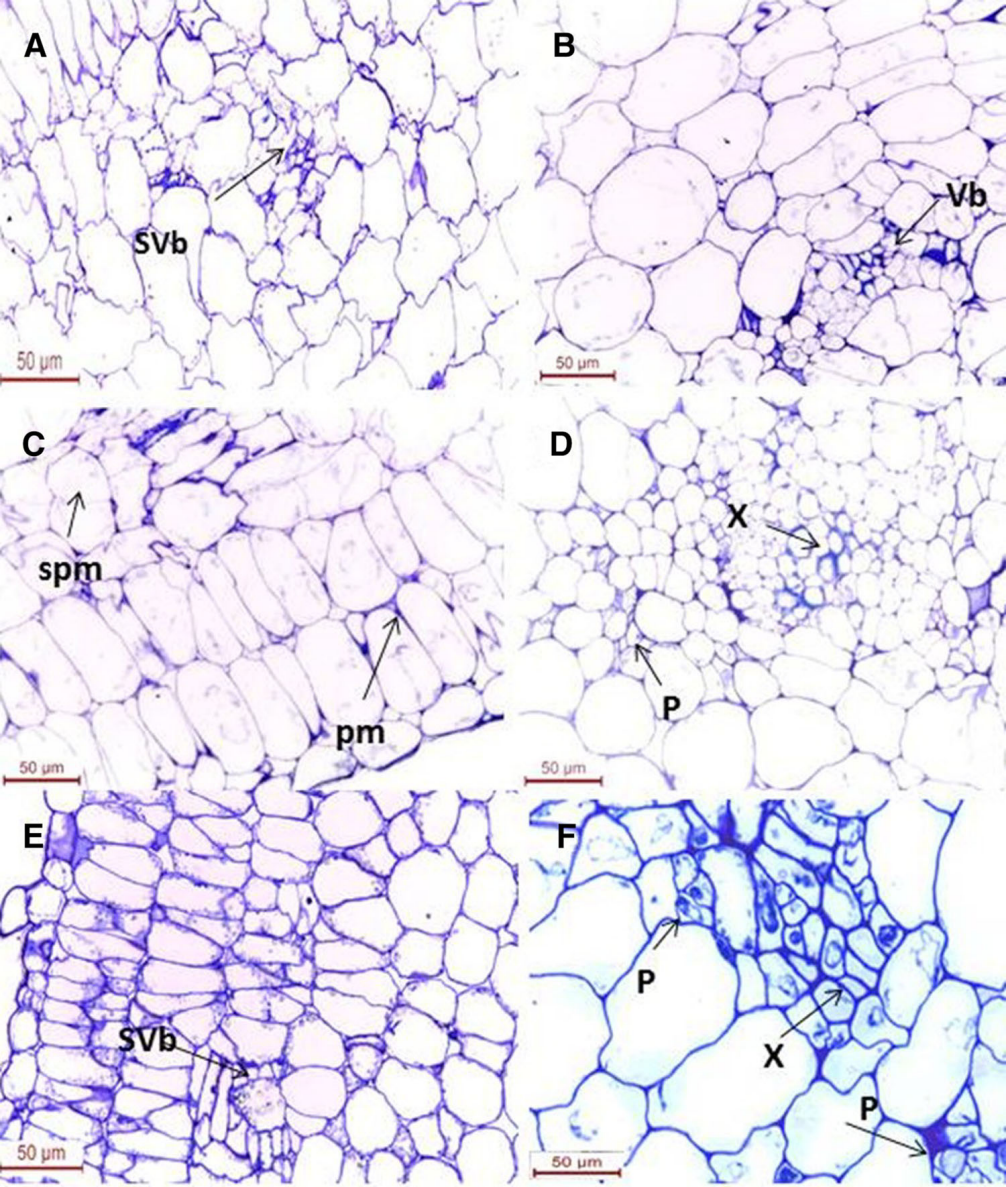
Histochemical localization of GUS transient expression. **a** pCambia3301. **b**, **c** pMMPTY. **d** pTMPTY. **e** pMMPWm. **f** pTMPWm. All sections are transverse leaf sections of transformed tomato explants. *vb* vascular bundle, *x* xylem, *p* phloem, *ep* external phloem, *ip* internal phloem, *v* vein, *pm* palisade mesophyll, *sm* spongy mesophyll, *svb* secondary vascular bundle. *Bar* 10, 20, 100 µm

To determine the expression levels of each putative promoter, tomato, watermelon, and rice were infiltrated with the five prepared constructs. Histochemical GUS assays were performed 3 days after *Agrobacterium* infiltration. The assays revealed different intensities of blue GUS activity among the TMPTY-pro, MMPTY-pro, TMPWm-pro, MMPWm-pro, and e35S-pro constructs in all tested tissues, while CPTY-pro yielded no GUS activity. MMPTY-pro, the minor fragment of the MP promoter of TYLCV, showed the highest intensity blue colour. The expression pattern of each putative promoter was monitored in transverse sections of infiltrated tomato tissues. Blue staining with MMPTY-pro was observed clearly in vascular bundle as well as the palisade mesophyll and spongy mesophyll (Fig. 5b, c). MMPWm-pro showed the next highest intensity of GUS activity, with blue staining in xylem and phloem cells and in secondary vascular bundles (Fig. 5e). Expression of TMPTY-pro and TMPWm-pro was detected in mature xylem and phloem cells of the infiltrated tomato tissues (Fig. 5d, f), with a similar intensity to the e35S promoter (Fig. 5a). Although CPTY-pro harboured the most *cis*-acting elements within its length, it did not promote GUS expression in any of the plants.

### Quantitative GUS assay

**Fig. 6:**
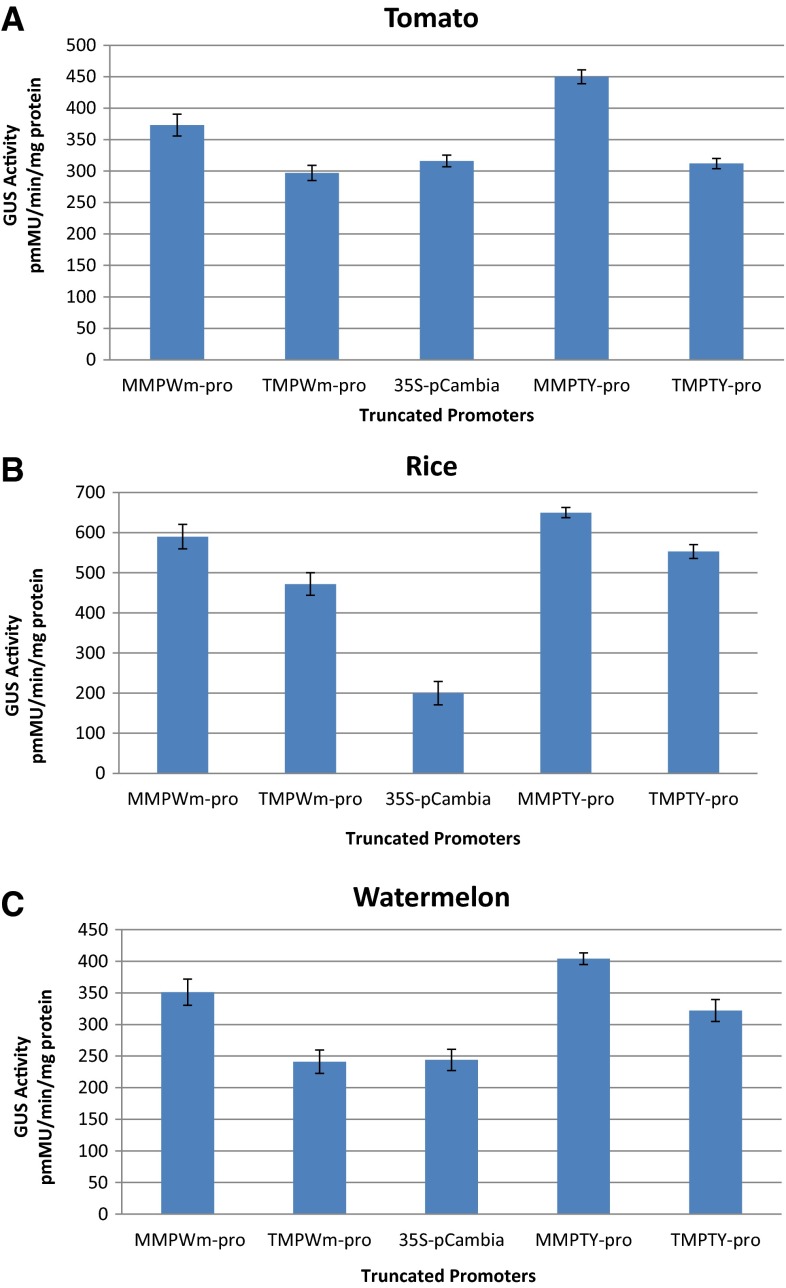
Quantitative fluorometric assay for the truncated promoters in **a** tomato, the main host for TYLCV; **b** watermelon, the main host for WmCSV; and **c** rice, a monocot

To precisely determine the expression level of each putative promoter, quantitative fluorometric assays were done to measure promoter efficiency in driving *GUS* in tomato, watermelon, and rice. Five replicates of each promoter in each type of explant were subjected to this assay. 3 days post-agroinfiltration, the mean values of the replicates were calculated and plotted (Fig. 6). CPTY-pro was not analysed because it showed no activity during the histochemical GUS assay.

In general, MMPTY-pro and MMPWm-pro had higher GUS expression levels than the longer fragments TMPTY-pro and TMPWm-pro in both dicot and monocot plants. Moreover, the minor fragment MMPTY-pro showed higher expression than the minor fragment MMPWm-pro. The e35S promoter activity was lowest among the putative promoters in the monocot plant.

In tomato, which is the main host of TYLCV, the highest GUS activity was driven by the truncated promoter MMPTY, followed by MMPWm-pro, then e35S-pro and TMPTY-pro (with nearly equal activities), and finally TMPWm-pro (Fig. 6a). In watermelon, the main host of WmCSV, GUS had the highest level of expression when driven by MMPTY-pro, followed by MMPWm-pro, TMPTY-pro, and TMPWm-pro and e35S (with nearly identical levels; Fig. 6b). Rice was used to evaluate these promoters in monocots; GUS assays indicated that the highest level of expression was obtained when the gene was controlled by MMPTY-pro, followed by MMPWm-pro, TMPTY-pro, TMPWm-pro, and finally e35S (Fig. 6c).

## Discussion

Promoters are regulatory regions that control gene expression in eukaryotes, which require exceptionally precise systems to regulate complex expression patterns involving thousands of genes (McKenna and O’Malley 2002a, b; Narlikar et al. 2002; Orphanides and Reinberg 2002). The promoters of plant viruses have great potential for plant biotechnology to engineer recombinant proteins with higher yields rapidly and at large scales (Pogue et al. 2002). Viruses such as *Potato virus X*, *Tobacco mosaic virus* (TMV), *Alfalfa mosaic virus*, *Cucumber mosaic virus*, and *Cowpea mosaic virus* have been used as expression vectors to produce recombinant proteins, like vaccine antigens (Donson et al. 1991; Chapman et al. 1992; Yusibov et al. 1997; Lomonossoff and Hamilton 1999; Zhao et al. 2000; Sanchez-Navarro et al. 2001; Pogue et al. 2002; Yusibov and Rabindran 2004).

The more basic knowledge we have on fundamental transcription elements in active promoters, the more efficient in vitro transcription we can perform in the future. Plant promoters that are activated precisely when and where needed would be ideal for genetic engineering. Therefore, the isolation and characterization of new effective functional plant promoters is highly desirable. To advance this goal, five putative promoter regions were isolated from two geminiviruses with bidirectional gene transcription from a stretch of DNA containing the core promoter region.

By comparing the different *cis*-elements present in each of our putative promoters and the promoters’ quantitative fluorescent GUS assays, we were able to make some valuable observations, which will significantly enhance future research on promoter architectures and activities. MMPTY-pro, which conferred the highest level of GUS expression, had the fewest conserved motifs (26 *cis*-acting elements), followed by MMPWm-pro (31). Although CPTY-Pro had the most elements, it did not promote expression. Interestingly, although CPTY-pro is a TATA-less promoter, this fact did not explain its behaviour, this observation is consistent with the observation of Khan et al. 2015) when it has observed a weak activity of CLCuBuV CP promoter which has been explained to probably be due to the absence of *AC2* gene product in CLCuBuV (Amrao et al. 2010). The role of transactivator AC2 protein for activation of virion sense promoter which has already been reported (Hong et al. 1996 and Sunter et al. 1990) and may have a great effect on the activity of CP promoter. Gene expression process in geminivirus happens by an early expression of complimentary sense gene, whose products are then participating in viral replication such as *AC1* (Rep) or they may act as transcription activator for the virion sense gene expression, such as *AC2* (TrAp). In contrast, the expression of virion sense genes usually appears later and requires complimentary sense gene product/products for activation (Ashraf et al. 2014). We have observed that the MMPTY-pro, which had the highest expression levels, was TATA-less like the CP which means that the TATA box had no great effect on the promoter activity in our case.

TATABOXes (TATABOX4, TATABOX5, TATABOXOSPAL) comprised 2 % of e35S-pro, 16 % of MMPWm-pro, 10 % of TMPWm-pro, and 8 % of TMPTY-pro. With TATABOX-dependent core promoters, the transcription factors (TFs) can assemble into a pre-initiation complex in the following order: TFIID, TFIIB, RNA polymerase II-TFIIF complex, TFIIF, and then TFIIH. TFIID consists of TBP (TATA box-binding protein) and about 13 TBP-associated factors (Burley and Roeder 1996; Albright and Tjian 2000; Berk 2000; Verrijzer 2001; Tora 2002), while TFIIB is single polypeptide that interacts with TBP as well as DNA upstream of the TATA box. In TATA-less promoters (e.g. CPTY-pro and MMPTY-pro), DPE is the downstream core promoter binding site of TFIID (Burke and Kadonaga 1996).

Remarkably the brightest blue colour of GUS promoted by MMPTY-pro, the TATA-less promoter, was observed constitutively in vascular bundles, spongy mesophyll, palisade mesophyll, and in the green plastids within palisade cells of tomato leaves. This result agrees with Erb and van Nimwegen (2011), who stated that TATA-less promoters are expressed in a constitutive manner and enriched for house-keeping genes, whereas TATA-containing promoters show variability in expression, and are often induced in response to stress. Our other putative promoters showed different expression patterns within plant tissues. The GUS staining derived from pMMPWm, with the second most intense levels, occurred in mature xylem and phloem, as well as in secondary vascular bundles. This staining was restricted to mature xylem and phloem in pTMPTY, pTMPWm, and pCAMBIA3301.

The activity results proved that the smaller fragments of the promoters were more efficient than their larger corresponding fragments. Comparing both activity level and the *cis*-acting elements present within the small MMPTY-pro and the large TMPTY-pro, we found that ARR1AT, GT1CONSENSUS, GTGANTG10, ACGTATERD1, WRKY71OS, EECCRCAH1, and ASF1MOTIFCAMV comprised 15, 7, 7, 4, 7, 7, and 4 %, respectively, of MMPTY-pro and 8, 5, 3, 3, 3, 3, and 2 %, respectively, of TMPTY-pro. In the WmCSV promoter fragments, TATABOX5, ARR1AT, CAATBOX1, CACFTPPCA1, ARFAT, and ASF1MOTIFCAMV made up 10, 10, 10, 10, 6, and 6 %, respectively, of the small MMPWm-pro and 8, 8, 8, 8, 4, and 4 %, respectively, of the large TMPWm-pro. Therefore, we postulate that the frequency of some *cis*-acting elements within the promoter region plays a crucial role in the promoter’s efficiency.

In this study, ARR1AT BOX, which comprised 15 % of MMPTY-pro and 8 % of TMPTY-pro, may have had a positive effect on the promoter’s efficiency. This motif made up 10 % of MMPWm-pro and 8 % of TMPWm-pro. ARR1 belongs to the MYB TF family and is one of seven members in the largest subclass of type-B ARRs (Sakai et al. 2000; Imamura et al. 2003; Tajima et al. 2004; Taniguchi et al. 2007). The MYP TF family is present in all eukaryotes. All members have the N-terminus MYP DNA-binding domain (Nero et al. 2009). ARR1 mediates cytokinin signal responses (Sakai et al. 2000, 2001; Hwang and Sheen 2001). Therefore, increasing the frequency of the ARR1AT BOX within our putative truncated promoters map explain the elevated GUS activity in both of our minor fragment promoters. Because BAP was used as a cytokinin source in all of our tissue cultures, it acted as a positive signal that enhanced the response of the ARR1 DNA-binding domain to the ARR1AT BOX. Consequently, the GUS expression was considerably increased by the truncated putative promoters.

ASF1MOTIFCAMV is another element that made up a greater percentage of the promoter region in both MMPTY-pro (4 %) and MMPWm-pro (6 %) compared with TMPTY-pro (2 %) and TMPWm-pro (4 %), respectively. This motif comprised 4 % of the e35S promoter and reacts with the ASF-1 cellular factor (Lam et al. 1989).

Another element, WRKY71OS, is a member of the W-BOX. WRKY is one of the largest transcriptional regulator families in the plant kingdom and is considered as an essential part of the signalling pathways that moderates many plant processes. WRKYs are proposed to perform a regulatory role in resistance transcriptome amid the W-box element [(C/T)TGAC(C/T] (Eulgem and Somssich 2007), and an enormous number of WRKY genes are up-regulated by pathogen infection (Maleck et al. 2000; Dong et al. 2003; Glazebrook et al. 2003; Kalde et al. 2003; Eulgem and Somssich 2007). Specific WRKY proteins have been proven to regulate plant immune responses. *Arabidopsis* WRKY52/RRS1 helps in defence against the *Ralstonia solanacearum* bacterium. Another *Arabidopsis* WRKY70 acts as a positive regulator of salicylic acid-dependent defence mechanisms (Li et al. 2004, 2006). Therefore, elevating the frequency of WRKY1OS in MMPTY-pro may be another mechanism to enhance GUS expression, because the *Agrobacterium* that we used to introduce our constructs into the explants is considered to be a biotic stress that can induce the WRKY TFs to initiate immune signalling cascades by binding to their proper element, in this case WRKY1OS.

In contrast, our analysis found that MYBCORATCYCB, MYCCONSESUSAT, MYB2CONSENSHSA MYBCORE, POLLEN1LELAT52, SEF1MOTIF, LEAFYATAG, LECPLEACS2, and POLASIG2 had been completely deleted from MMPTY-pro, while MYCCONSESUSAT, POLLEN1LELAT52, POLASIG1, GTGANTG10, and GT1CONSENSUS had been completely deleted from MMPWm-pro, although they were present in the longer versions of these promoters. This result suggested that the deletion of some elements could decrease the competition of different TFs for their specific elements within the promoter region.

The five conserved domains ARR1AT, CAATBOX1, ACGTATERD1, GATABOX, and DOFCOREZM were common in all of our putative promoters and in the 35S promoter. CAATBOX1 occupies a considerable proportion of our putative promoter fragments: 10 % in MMWm-pro, 9 % in CPTY-pro, 8 % in TMPWm-pro and TMPTY-pro, 7 % in MMPTY-pro, and finally 5 % within e35S. This motif is responsible for the tissue-specific promotion of the pea legumin gene *LegA* (Shirsat et al. 1989) and occurs within the pvPDF promoter. The ACGTATERD1 domain is required for etiolation-induced expression of *erd1* (early response to dehydration) in *Arabidopsis*. GATABOXes are required for high-level light-regulated and tissue-specific gene expression. GATA TFs are a group of DNA-binding proteins distinguished by a zinc finger motif that have been implicated in light- and nitrate-dependent transcription control (Reyes et al. 2004). GATA TFs are reported to bind the CaMV 35S promoter and are conserved in cab promoters as well (Lam et al. 1989). DOFCOREZM is the target binding site of Dof proteins, which are specific DNA-binding proteins associated with the expression of multiple genes in plants. Dof proteins also differentially regulate diverse promoters in a variety of plant tissues (Yanagisawa and Schmidt 1999).

Gene expression, and thus protein biosynthesis, does not depend only on the presence of functional *cis*-elements, but also on how and when the *trans*-acting elements interact with their proper elements and also on the activity of RNA polymerase II. The transcriptome activation of CPTY-pro is mediated by transactivation of the C2, which might explain the silent expression of this putative promoter.

## Conclusions

The information that we have elucidated on the begomoviral promoter of TYLCV and WmCSV, a very distinct Old World and newly emerged begomovirus, would help in further elucidating transcription regulation in begomoviruses. Thus, generating more efficient tools in producing transgenes.

ARR1AT, GT1CONSENSUS, GTGANTG10, ACGTATERD1, WRKY71OS, EECCRCAH1, ASF1MOTIFCAMV, TATABOX5, CAATBOX1, CACFTPPCA1 and ARFAT might be strong candidates for enhancing transcription. The constitutive expression profile of MMPTY-pro can be explained by the fact that it is a TATA-less promoter. In future work, we intend to investigate the capability of these recommended motifs to enhance promoter activity for large-scale in vitro protein production.
